# A Multilevel Approach to Breastfeeding Promotion: Using Healthy Start to Deliver Individual Support and Drive Collective Impact

**DOI:** 10.1007/s10995-017-2371-3

**Published:** 2017-11-22

**Authors:** Chelsey Leruth, Jacqueline Goodman, Brian Bragg, Dara Gray

**Affiliations:** Access Community Health Network, 600 W Fulton St, Suite 200, Chicago, IL 60661 USA

**Keywords:** Breastfeeding, Breastfeeding support, Community health

## Abstract

*Purpose* Breastfeeding has been linked to a host of positive health effects for women and children. However, disparities in breastfeeding initiation and duration prevent many low-income and African-American women from realizing these benefits. Existing breastfeeding promotion efforts often do not reach women who need support the most. In response, the Westside Healthy Start program (WHS), located in Chicago, Illinois, developed an ongoing multilevel approach to breastfeeding promotion. *Description* Key elements of our WHS breastfeeding model include individual education and counseling from pregnancy to 6 months postpartum and partnership with a local safety-net hospital to implement the Baby-Friendly Hospital Initiative and provide lactation support to delivering patients. *Assessment* In the year our model was implemented, 44.6% (49/110) of prenatal WHS participants reported that they planned to breastfeed, and 67.0% (183/273) of delivered participants initiated. Among participants reaching 6 months postpartum, 10.5% (9/86) were breastfeeding. WHS also had 2667 encounters with women delivering at our partner hospital during breastfeeding rounds, with 65.1% of contacts initiating. Community data was not available to assess the efficacy of our model at the local level. However, WHS participants fared better than all delivering patients at our partner hospital, where 65.0% initiated in 2015. *Conclusion* Healthy Start programs are a promising vehicle to improve breastfeeding initiation at the individual and community level. Additional evaluation is necessary to understand barriers to duration and services needed for this population.

## Significance

*What is already known on this subject?* Low-income women and racial/ethnic minorities face distinct and numerous difficulties that contribute to persistent disparities in breastfeeding initiation and duration rates. Comprehensive breastfeeding information and support is not reliably available to these populations.

*What this study adds?* This evidence-informed model provided individual and systems-level breastfeeding support to low-income, African-American women throughout pregnancy and the postpartum period. Results offer evidence that this model has improved breastfeeding rates for participants.

## Purpose

Breastfeeding has tremendous benefits for women and children. Breastfed children have lower risk of sudden infant death syndrome, common childhood infections, asthma, diabetes, and childhood obesity. Women who nurse have reduced risk of breast and ovarian cancer. Breastfeeding promotes mother-infant bonding and may protect against postpartum depression (U.S. Department of Health and Human Services 2011).

Despite overall improvement in breastfeeding rates, persistent disparities prevent many low-income and African-American women from realizing these benefits. Nationwide, 83% of White women initiated breastfeeding in 2012 compared to 66% of African-American women. Mothers with lower income and education levels were also less likely to initiate. These disparities widen during the infant’s first year. For example, at 6 months 56% of White women breastfed compared to 35% of African-American women (Centers for Disease Control and Prevention 2015).

All women experience breastfeeding challenges, but low-income women and racial/ethnic minorities face distinct and more numerous difficulties including lack of support at home, work, and in their communities, language and literacy barriers, increased tobacco and alcohol use, and insufficient information about breastfeeding benefits and techniques (Jones et al. 2015). Lack of appropriate support from health and social service providers is a particular problem. For example, a study of infant feeding intentions among low-income pregnant women found that less than a quarter had received breastfeeding information from a health professional (Gurka et al. 2014). Even more troubling, African-American women have reported differential treatment regarding breastfeeding support from health providers (Ringel-Kulka et al. 2011). Historically, the availability of free formula from WIC has discouraged breastfeeding (Jensen 2012). However, over the past decade WIC implemented changes such as revised food packages and breastfeeding peer counselor programs that have been associated with improved breastfeeding rates (Special Supplemental Nutrition Program for WIC 2014; May et al. 2015).

The National Healthy Start (NHS) program funded by the Health Resources and Services Administration is an ideal vehicle to deliver breastfeeding support to women disproportionately less likely to nurse. NHS has tremendous reach, serving some of the nation’s poorest and most at-risk families in 87 communities nationwide (Health Resources and Services Administration n.d.-b). NHS projects must include evidence-informed practices to promote breastfeeding initiation and duration (Health Resources and Services Administration n.d.-a).

Our local Westside Healthy Start program (WHS) has a 19-year history of working to improve perinatal health for high-risk, predominantly African-American families living in Chicago, Illinois. Our WHS communities have an infant mortality rate of 14.1 per 1000 live births, more than double the national figure (Illinois Department of Public Health 2014), and bear the weight of poverty, unemployment, and low educational attainment. Our WHS program is led by a federally qualified health center organization certified as a patient-centered medical home by the National Committee for Quality Assurance. Key WHS services include community outreach and education, case management for 800 families annually, and male involvement.

Available data point to low breastfeeding rates in our WHS project area. Among low-income African-American women across Illinois (a similar demographic to WHS program participants), 52% initiated breastfeeding in 2004–2008 (HealthConnect, & One, Illinois Department of Human Services, & University of Illinois at Chicago 2011). In 2015, 65.0% of newborns at a safety-net hospital in the WHS project area were breastfed (Illinois Department of Public Health n.d.). To address breastfeeding disparities among our target population and fulfill the national program mission, our WHS program developed and implemented a unique model to promote breastfeeding initiation and duration by creating systems, policies, and practices that support nursing and decrease barriers.

## Description

### Program Planning

To develop a model for breastfeeding promotion, we consulted previous WHS program data and breastfeeding promotion literature. Two resources greatly informed our approach: the Illinois Breastfeeding Blueprint (HealthConnect, & One, Illinois Department of Human Services, & University of Illinois at Chicago 2011) and the Guide to Breastfeeding Interventions (Centers for Disease Control and Prevention 2013). Both tools emphasize early, frequent intervention on multiple levels, which we incorporated into our model.

Our evidence-informed breastfeeding support model is unique in its combination of ongoing individual support and a systems-level approach. First, our model serves women continuously from pregnancy through the postpartum period. Services are timed to crucial moments for breastfeeding success: during pregnancy to address knowledge and intentions, at delivery to promote timely initiation, and postpartum to encourage maintenance. Second, individual services are complemented by collaboration with a local hospital to improve systems of care. WHS breastfeeding services are delivered by breastfeeding support counselors (BFCs), certified lactation counselors who received training in this new model, described below.

### Prenatal Education and Counseling

All WHS participants receive general breastfeeding education from case managers throughout pregnancy (such as information about breastfeeding benefits) and one face-to-face visit from a BFC in the third trimester for more personalized and comprehensive support. The prenatal BFC visit takes place at the WHS program site or the participant’s home for about 30–60 min. BFCs follow a standard protocol based on Best Start Three Step Counseling, designed to engage women ‘where they are’ before delivery (Bryant and Roy 1997). Each prenatal BFC visit includes: (1) open-ended questions to elicit breastfeeding beliefs, (2) affirmation of participant feelings and concerns, and (3) targeted education. The BFC also discusses breastfeeding intentions, describes breastfeeding benefits, teaches positioning, and facilitates ordering a breast pump if desired.

### Hospital Collaboration and Delivery Support

WHS collaborates with the largest delivering hospital in our service area, a safety-net provider, to improve the breastfeeding environment and systems of care. This collaboration addresses four conditions of collective impact, a key strategy of the NHS program (Kania and Kramer 2011):

1. Common agenda—WHS promotes and advises on the hospital’s pursuit of Baby-Friendly Hospital designation (World Health Organization and UNICEF 2009). The lead WHS organization and the hospital have a longstanding administrative and clinical partnership that serves as the foundation for breastfeeding collaboration.
2. Shared measurement—breastfeeding initiation data is consistently collected and monitored as a WHS benchmark and an indicator on the Illinois hospital report card.
3. Continuous communication—since 2010, a WHS program manager has participated in the hospital’s breastfeeding multidisciplinary committee.
4. Mutually reinforcing activities—by providing consultation and lessons learned from serving patients, WHS has assisted the hospital to implement breastfeeding-friendly policies, training, and practices. By the end of 2015, the hospital had reached the second phase (development) of the pathway to Baby-Friendly designation and had implemented key practices such as skin-to-skin immediately following delivery and no formula distribution or pacifier use. WHS also provides staff to enhance hospital capacity for breastfeeding support. WHS BFCs conduct weekday rounds alongside the hospital’s part-time International Board Certified Lactation Consultants on the mother/baby unit. The BFC follows a standard workflow that includes education on breastfeeding benefits, safe sleep, and well visits; coaching interested women on positioning; and referrals to reduce barriers (e.g. breast pumps, WIC, and behavioral health). By request, the BFC provides consultation in obstetrics triage, labor and delivery, and the neonatal intensive care unit. BFC hospital encounters ranges from 5 to 20 min.

### Postpartum Follow-Up

After initiating, WHS participants receive BFC contact for 6 months and general support from their case manager while remaining in the program. Postpartum support aims to help women continue breastfeeding through encouragement, management of lactation crises, and planning for transitions back to work or school. BFC contact is more frequent immediately after delivery when women are most likely to stop nursing; contact is weekly from delivery to 3 weeks, bi-weekly from 1 to 3 months, and monthly from 4 to 6 months. Most BFC postpartum contact is by telephone, ranging from 5 to 10 minutes. By participant request, BFCs also conduct home visits or meet in-person at a WHS site for about 30–60 min.

### Data Collection and Analysis

WHS participants sign a consent form describing program services and data collection procedures. Results presented here only represent participants who agreed to include their data in public reports. In May 2015, WHS began collecting data on program services, participant demographics, health beliefs, and outcomes through REDCap, a secure web-based data collection system (Harris et al. 2009). REDCap’s customized data entry forms and live reports enable WHS staff to coordinate breastfeeding services across program sites. BFCs also document aggregate hospital round data in an online spreadsheet.

WHS data were exported from REDCap, and then cleaned, recoded, and analyzed in SPSS. Descriptive statistics were used to assess demographics, services, and health outcomes. Breastfeeding initiation was calculated for WHS participants with a single or multiple live birth. Breastfeeding duration was calculated for infants reaching 6 months in WHS. Participants with unknown breastfeeding status were excluded. Qualitative data were exported to Microsoft Excel for analysis. Participant comments were labeled with codes for key ideas. Encounter notes were reviewed for fidelity to program protocols. All WHS results are for calendar year 2015 unless otherwise noted.

## Assessment

In 2015, WHS served 651 women participants. As seen in Table 1, most participants were non-Hispanic, African-American women under age 25. Additional demographics available for a subset of women enrolled since REDCap implementation indicate that participants were largely low-income, Medicaid recipients with a high school diploma.

**Table 1:** Characteristics of 2015 WHS Participants

	N = 651	%
Age		
< 17	52	8.0
18–24	320	49.2
25–34	243	37.3
35–45	36	5.5
Race		
Black or African American	624	95.9
White	19	2.9
Native Hawaiian or Pacific Islander	1	0.2
Multiracial	1	0.2
Unknown	6	0.9
Ethnicity		
Not Hispanic or Latino	623	95.7
Hispanic or Latino	27	4.1
Unknown	1	0.2
Federal poverty level (FPL)		
< 100% FPL	541	83.1
101–185% FPL	21	3.2
> 185% FPL	2	0.3
Unknown	87	13.4
Insurance status		
Medicaid	513	78.8
Private	12	1.8
None	3	0.5
Unknown	123	18.9
Educational attainment		
12 or fewer years of school	117	18.0
High school diploma or GED	167	25.7
Some college	88	13.5
Unknown	279	42.9

**Fig. 1:**
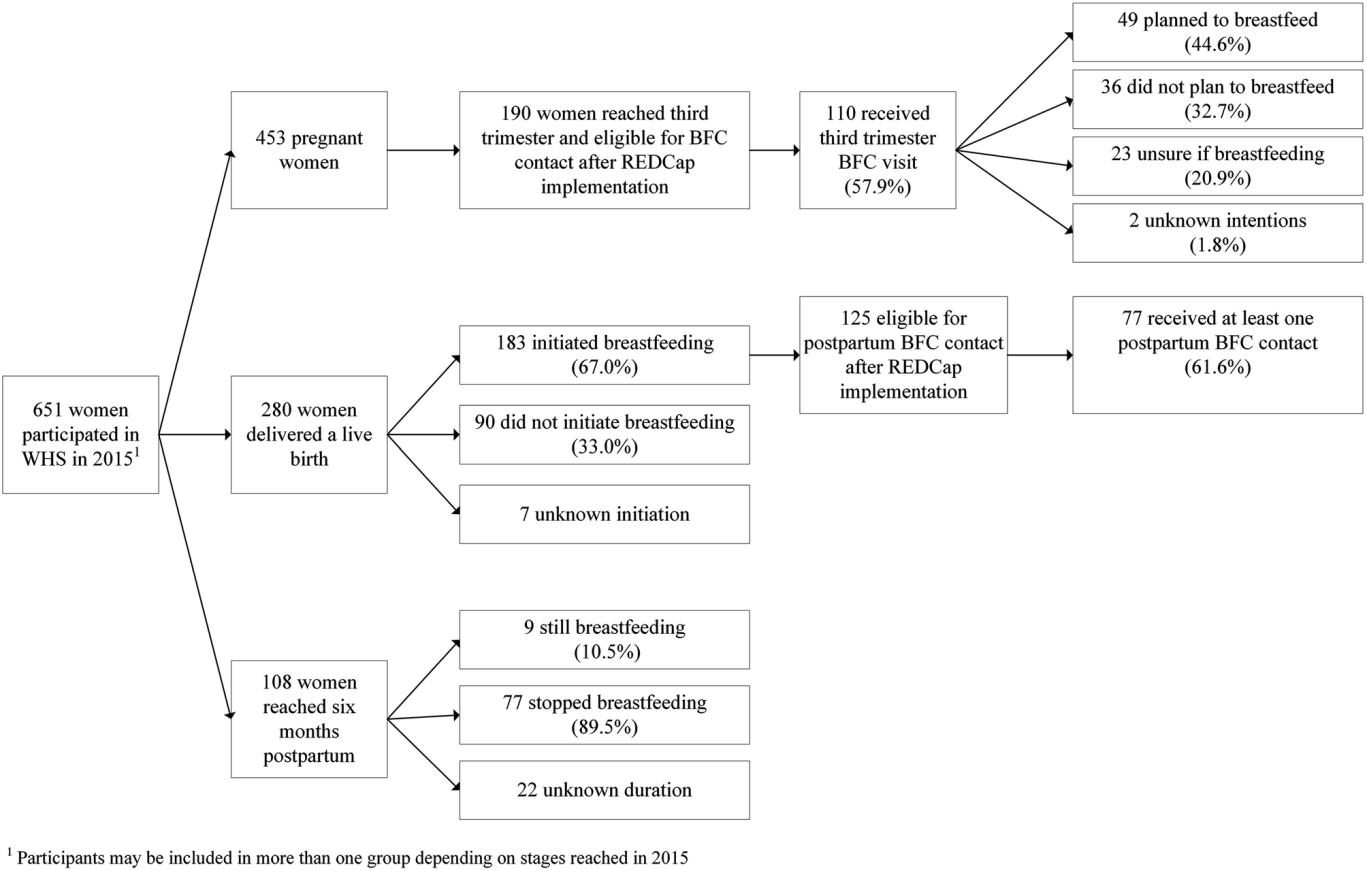
Breastfeeding services, attitudes, and outcomes among WHS participants

Breastfeeding services, intentions, and outcomes for three types of WHS participants (pregnant, delivered, and postpartum) are summarized in Fig. 1 and described below. Participants may be included in more than one group depending on perinatal stages reached in 2015.

### Prenatal Breastfeeding Beliefs and Intentions

Prenatal encounter notes indicate BFCs provided targeted affirmation and guidance to participants, discussing breastfeeding knowledge, fears, and available resources. BFCs used key statements such as “[breastfeeding] is different for every individual” and “help is available at [the] hospital and clinic to her as many times as needed.” BFCs also educated participants on latching, positioning, feeding cues, feeding frequency, and proper hydration and nutrition while breastfeeding. Data available for May–December 2015 (after REDCap implementation) showed that 57.9% of eligible women (110/190) received a third trimester BFC visit. On average, visits occurred at 35 weeks gestation (range 29–39 weeks). At that visit, 44.6% of participants (n = 49) reported that they planned to breastfeed, 20.9% (n = 23) were unsure, and 32.7% (n = 36) did not plan to breastfeed (two participants did not report breastfeeding intentions). When asked “What have you heard about breastfeeding?”, the most common responses related to breastfeeding benefits and discomfort. Other responses are displayed in Table 2.

**Table 2:** Views on breastfeeding at third trimester visit

What have you heard about breastfeeding?	n = 110
Breastfeeding is best, healthy, or beneficial option for mother and/or baby	40.0% (n = 44)
Breastfeeding is “painful” or “uncomfortable”	18.2% (n = 20)
“Nothing” or “unsure”	11.8% (n = 13)
Do not plan to breastfeed	10.9% (n = 12)
Have breastfed before	4.5% (n = 5)
Have breastfed before and it was a bad experience	4.5% (n = 5)
Cannot breastfeed (smoking, HIV status)	2.7% (n = 3)
“No one in my family did it”	0.9% (n = 1)
“[Breastfed] babies get spoiled”	0.9% (n = 1)
No response	5.5% (n = 6)

### Breastfeeding Initiation

Among WHS participants with a single or multiple live birth in 2015, 67.0% (183/273) initiated breastfeeding. Initiation was unknown for seven participants. WHS also had 2667 encounters with women delivering at our partner hospital during breastfeeding rounds, with 65.1% (n = 1735) of contacts initiating.

### Breastfeeding Duration

**Fig. 2:**
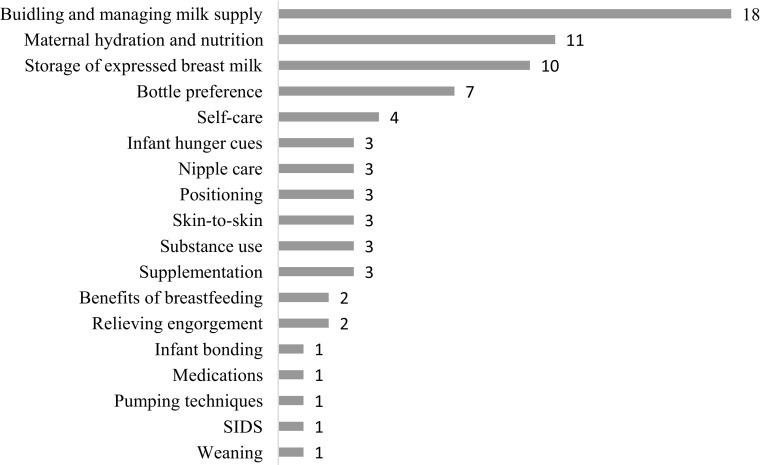
Count of reasons WHS participants stopped breastfeeding

At 6 months, 10.5% (n = 9/86) of WHS infants were breastfeeding. Breastfeeding status at 6 months was unknown for 22 participants. Among women who initiated breastfeeding between May and December 2015 (after REDCap implementation), 61.6% (n = 77/125) received at least one postpartum visit from a BFC. The most common breastfeeding issues reported by participants were supply problems and nipple pain (see Table 3). Other issues identified included engorgement, smoking, and substance use. As seen in Fig. 2, BFCs provided education on a variety of topics and most frequently discussed building and managing milk supply (18 encounters), maternal hydration and nutrition (11), and storage of expressed breast milk (10). BFCs referred ten participants for breast pumps.

**Table 3:** Reported breastfeeding issues at postpartum contacts

Postpartum breastfeeding issues	n = 71
Breast milk supply issue	25.4% (n = 18)
Sore nipples	21.3% (n = 15)
Latch issue	16.9% (n = 12)
Pumping issue	14.1% (n = 10)
Other issue	22.5% (n = 16)

**Fig. 3:**
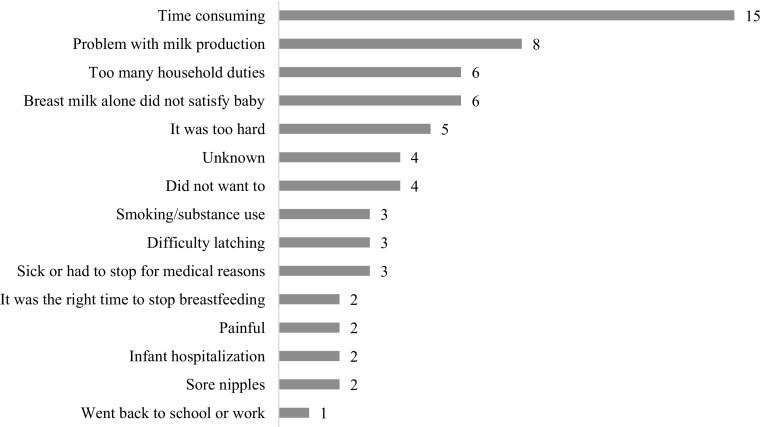
Count of education topics in postpartum WHS BFC encounters

Participants who received postpartum BFC contact and stopped breastfeeding between May and December 2015 had an average of two but as many as eight direct contacts with a BFC. Figure 3 shows a count of all the reasons participants reported for stopping breastfeeding (multiple responses were allowed). The most common reason was that breastfeeding was “too time consuming.”

## Conclusion

By intervening on multiple levels before, during, and after delivery, this model maximizes the opportunities to improve breastfeeding rates among WHS participants and community members.

### Breastfeeding Initiation

Program results and comparison data provide evidence that this comprehensive approach improves breastfeeding initiation for WHS participants. In 2013 (the last year before this model was implemented), 58.5% (113/193) of WHS participants initiated breastfeeding compared to 67.0% in 2015 when our new model was deployed. WHS participants also fared better than a similar demographic of low-income African-American women across Illinois, 52% of whom initiated breastfeeding in 2004–2008.

Challenges with implementing new services, scheduling, and reaching participants prevented about one-third of eligible women from receiving a prenatal BFC visit. Data on refusals is not available, but some participants may have declined to meet with the BFC. Since over half of participants have already decided not to breastfeed by the third trimester, intervening earlier during pregnancy may be beneficial.

### Hospital Environment of Care

Hospital collaboration has extended the reach of WHS services to the broader community and contributed to collective impact. The rate of breastfeeding initiation was similar among women who received rounds from a WHS BFC (65.1%) compared to data reported by the partner hospital (65.0%), suggesting that WHS BFCs reached a majority of delivering hospital patients. By providing consultation and staff resources, WHS has supported the progress of our partner hospital towards achieving Baby-Friendly status and helped to create environmental conditions friendly to breastfeeding at the critical time immediately after delivery.

### Breastfeeding Duration

WHS breastfeeding rates at 6 months have improved since the implementation of our new model, from 6.2% (9/145) in the last budget year of the previous grant cycle (June 2013–August 2014) to 10.5% in calendar year 2015. However duration remains an area for improvement; considerable progress is needed to reach NHS and Healthy People 2020 goals.

Additional investigation is needed to understand which factors pose the greatest barriers to duration and how they interact for this population. WHS participants reported that time demands were their main obstacle to continued breastfeeding. This differs from all Illinois women, who most commonly cited problems with milk production and breast milk alone not satisfying the infant (Illinois Department of Public Health 2012). In addition to individual support, structural changes such as improved maternity leave policies and economic equality (U.S. Department of Health and Human Services 2011) may increase breastfeeding duration rates for this population. Participant comments such as “I want to try to breastfeed [but] everyone around me has said…that it hurts,” indicate that negative community attitudes towards breastfeeding are also a significant barrier. Our WHS program is developing plans to address several of these factors including a social marketing campaign to improve community breastfeeding norms, advocacy materials for participants and community members about nursing rights in workplaces and schools, and strategies to further involve male partners in breastfeeding education and support.

### Limitations

These results are limited by the observational evaluation design. Participation in WHS is voluntary, so participants and their outcomes may differ from other women. Community-level breastfeeding data are currently unavailable, precluding a local comparison of program efficacy. Additional limitations include potential history effects when comparing 2015 WHS results to earlier Illinois data, use of aggregate data for hospital rounds which may duplicate women receiving multiple encounters, and the large portion of WHS participants with unknown breastfeeding status at 6 months. Nevertheless, these results indicate our program is a promising approach for promoting breastfeeding among low-income African-American women.

## References

[CR1] Bryant C, Roy M (1997). Best start’s three-step counseling strategy.

[CR2] Centers for Disease Control and Prevention. (2013). *Strategies to prevent obesity and other chronic diseases: The CDC guide to strategies to support breastfeeding mothers and babies*. Retrieved from http://www.cdc.gov/breastfeeding/pdf/BF-Guide-508.pdf. 10.3945/an.114.005900 PMC4013182 24829476

[CR3] Centers for Disease Control and Prevention. (2015). Graph showing breastfeeding among U.S. Children Born 2002–2012. National Immunization Surveys. Retrieved from http://www.cdc.gov/breastfeeding/data/nis_data/index.htm.

[CR4] Gurka KK, Hornsby PP, Drake E, Mulvihill EM, Kinsey EN, Yitayew MS, Kellams AL (2014). Exploring intended infant feeding decisions among low-income women. Breastfeeding Medicine.

[CR5] Harris PA, Taylor R, Thielke R, Payne J, Gonzalez N, Conde JG (2009). Research electronic data capture (REDCap)A metadata-driven methodology and workflow process for providing translational research informatics support. Journal of Biomedical Informatics.

[CR6] Health Resources and Services Administration. (n.d.-a). Healthy Start grant program. Retrieved from http://mchb.hrsa.gov/programs/healthystart/grants/index.html.

[CR7] Health Resources and Services Administration. (n.d.-b). Maternal and child health discretionary grant information system. Retrieved from https://mchdata.hrsa.gov/dgisreports/ProgramData/ProgramReports.aspx?Report=PerinatalA.

[CR8] HealthConnect, & One, Illinois Department of Human Services, & University of Illinois at Chicago (2011). Illinois breastfeeding blueprint: A plan for change.

[CR9] Illinois Department of Public Health. (2012). [Table showing reported reasons for stopping breastfeeding]. Illinois PRAMS 2012. Retrieved from http://www.dph.illinois.gov/data-statistics/prams/datatables-2012. .

[CR10] Illinois Department of Public Health. (2014). Community area infant birth and death data for North Lawndale, East Garfield, West Garfield, Austin: 2007–2009 [Data file]. Special data request.

[CR11] Illinois Department of Public Health. (n.d.). *Illinois hospital report card and consumer guide to health care: Mount Sinai Hospital 2015*. Retrieved from http://www.healthcarereportcard.illinois.gov/hospitals/view/101162.

[CR12] Jensen E (2012). Participation in the supplemental nutrition program for Women, Infants and Children (WIC) and breastfeeding: National, regional, and state level analyses. Maternal and Child Health Journal.

[CR13] Jones KM, Power ML, Queenan JT, Schulkin J (2015). Racial and ethnic disparities in breastfeeding. Breastfeeding Medicine.

[CR14] Kania, J., & Kramer, M. (2011). Collective impact. *Stanford Social Innovation Review, 9*(1), 36–41. Retrieved from http://ssir.org/articles/entry/collective_impact.

[CR15] May L, Borger C, McNutt S, Harrison G, Weinfield N, MacAllum C, Montaquila J (2015). WIC infant and toddler feeding practices study 2: Intention to breastfeed.

[CR16] Ringel-Kulka T, Jensen E, Mclaurin S, Woods E, Kotch JB, Labbok M, Baker S (2011). Community based participatory research of breastfeeding disparities in African-American Women. ICAN: Infant, Child, & Adolescent Nutrition.

[CR17] Special Supplemental Nutrition Program for Women, Infants, and Children (WIC). (2014). Revisions in the WIC Food Package, 7 C.F.R. § 246. http://blog.apastyle.org/apastyle/2013/07/the-rules-for-federal-regulations-i-code-of-federal-regulations.html.

[CR18] U.S. Department of Health and Human Services. (2011). *The Surgeon General*’*s call to action to support breastfeeding*. Retrieved from http://www.surgeongeneral.gov/library/calls/breastfeeding/calltoactiontosupportbreastfeeding.pdf.

[CR19] World Health Organization & UNICEF. (2009). *Baby*-*friendly hospital initiative: Revised, updated and expanded for integrated care*. Retrieved from http://www.who.int/nutrition/publications/infantfeeding/bfhi_trainingcourse/en/. 23926623

